# Natural Compounds in Liposomal Nanoformulations of Potential Clinical Application in Glioblastoma

**DOI:** 10.3390/cancers14246222

**Published:** 2022-12-16

**Authors:** Ludwika Piwowarczyk, Dariusz T. Mlynarczyk, Violetta Krajka-Kuźniak, Aleksandra Majchrzak-Celińska, Anna Budzianowska, Szymon Tomczak, Jaromir Budzianowski, Aneta Woźniak-Braszak, Rafał Pietrzyk, Mikołaj Baranowski, Tomasz Goslinski, Anna Jelinska

**Affiliations:** 1 Chair and Department of Pharmaceutical Chemistry, Poznan University of Medical Sciences, Grunwaldzka 6, 60-780 Poznań, Poland; 2 Chair and Department of Chemical Technology of Drugs, Poznan University of Medical Sciences, Grunwaldzka 6, 60-780 Poznan, Poland; 3 Chair and Department of Pharmaceutical Biochemistry, Poznan University of Medical Sciences, Swięcickiego 4, 60-781 Poznan, Poland; 4 Laboratory of Pharmaceutical Biology and Biotechnology, Chair and Department of Practical Cosmetology and Prevention of Skin Diseases Prophylaxis, Poznan University of Medical Sciences, Rokietnicka 3, 60-806 Poznan, Poland; 5 Faculty of Physics, Adam Mickiewicz University in Poznan, Uniwersytetu Poznanskiego 2, 61-614 Poznan, Poland; 6 Novilet, Romana Maya 1, 61-371 Poznan, Poland

**Keywords:** cancer, glioblastoma, liposomes, natural compounds, drug delivery system

## Abstract

**Simple Summary:**

Glioma is a type of cancer that is the most common primary brain tumor in adults. The prognosis is often unfavorable despite early detection and treatment. Therefore, it is necessary to search for novel therapeutic approaches, new therapeutic agents and their delivery systems. Compounds derived from plants: orientin, actioside, curcumin, and bisdemethoxycurcumin, were put in liposomes. Then, the liposomes were tested for their anticancer activity on gliomas cell lines. It was found that all the compounds were active, with acteoside showing the highest activity. Additionally, a combination of the compounds was proven to be more active than the single components.

**Abstract:**

Glioblastoma (GBM) is the most common malignant neoplasm in adults among all CNS gliomas, with the 5-year survival rate being as low as 5%. Among nanocarriers, liposomal nanoformulations are considered as a promising tool for precise drug delivery. The herein presented study demonstrates the possibility of encapsulating four selected natural compounds (curcumin, bisdemethoxycurcumin, acteoside, and orientin) and their mixtures in cationic liposomal nanoformulation composed of two lipid types (DOTAP:POPC). In order to determine the physicochemical properties of the new drug carriers, specific measurements, including particle size, Zeta Potential, and PDI index, were applied. In addition, NMR and EPR studies were carried out for a more in-depth characterization of nanoparticles. Within biological research, the prepared formulations were evaluated on T98G and U-138 MG glioblastoma cell lines in vitro, as well as on a non-cancerous human lung fibroblast cell line (MRC-5) using the MTT test to determine their potential as anticancer agents. The highest activity was exhibited by liposome-entrapped acteoside towards the T98G cell line with IC_50_ equal 2.9 ± 0.9 µM after 24 hours of incubation. Noteworthy, curcumin and orientin mixture in liposomal formulation exhibited a synergistic effect against GBM. Moreover, the impact on the expression of apoptosis-associated proteins (p53 and Caspase-3) of acteoside as well as curcumin and orientin mixture, as the most potent agents, was assessed, showing nearly 40% increase as compared to control U-138 MG and T98G cells. It should be emphasized that a new and alternative method of extrusion of the studied liposomes was developed.

## 1. Introduction

Gliomas are the most common primary brain tumors in adults. They form a heterogeneous group of neoplasms whose incidence ranges from 1.9 to 9.6 per 100,000. Age, gender, ethnicity, and geography were found to be predisposing factors [1,2,3]. *The 2021 World Health Organization (WHO) Classification of Tumors of the Central Nervous System* distinguishes three types of adult-type diffuse gliomas, namely: astrocytoma (IDH-mutant); oligodendroglioma (IDH-mutant, and 1p/19q-codeleted); and glioblastoma (IDH-wildtype) [4]. The new classification takes into account not only histological parameters but also molecular differences. Although, according to this classification, all IDH-mutant diffuse astrocytic tumors are classified as astrocytoma, IDH-mutant (CNS WHO grade 2, 3, or 4), unlike astrocytoma, oligodendroglioma (IDH-mutant and 1p/19q-codeleted) is an infiltrating tumor and can be assigned to CSN WHO Grade 2 or 3. A typical location is the frontal lobe (approx. 60% of cases).

Glioblastoma (IDH-wildtype, GBM) is the most common malignant neoplasm in adults, which accounts for nearly 50% of all glioma cases. It is characterized by the presence of TERT promoter mutation, EGFR gene amplification, or  + 7/ −10 chromosome copy-number changes [5,6]. Moreover, if the GBM does not meet the specified criteria for WHO grade 4CNS, such as blood vessel proliferation and necrosis, but still manifests the above molecular abnormalities, it would then often develop a grade 4 malignant clinical course [7,8]. GBM is the most malignant and invasive CNS glioma accounting for 14.3% of all tumors and 49.1% of malignant tumors. Despite early detection and treatment, the prognosis is often unfavorable with the current median survival rates of patients showing no improvement at only eight months [9] proving that the current therapeutic options are still not satisfactory [10]. In population studies and according to the US Central Registry of Brain Tumors in 2014–2018, the 5-year survival rate for GBM did not exceed 5% [9]. The above data indicate a continuing need to search for new drugs and treatments for GBM.

Polyphenols constitute a large group of bioactive phytochemicals within the phenols group and contain at least two hydroxyl groups attached to an aromatic ring [11]. Curcuminoids belong to the group of polyphenols and are the most active substances in turmeric. This group of substances includes mainly curcumin (CUR) in 77%, demethoxycurcumin (DMC) in 17%, and bisdemethoxycurcumin (BDMC) in 3% [12]. Turmeric (*Curcuma longa*) belongs to the ginger family and is grown extensively in Southeast Asia. Turmeric rhizome is primarily used in Asian dishes as a spice to give food its yellow color, characteristic taste, and aroma. CUR is the main bioactive ingredient insulated with turmeric rhizomes, consisting of two feruloyl residues linked by a methylene group. It represents approximately 0.58% to 3.14% of the dry weight of turmeric [13]. Studies from recent years show that CUR expresses antioxidant, anti-inflammatory, antimicrobial, and anticancer properties. The problem associated with its wide clinical use is its low chemical stability and oral bioactivity. DMC and BDMC are naturally occurring analogs of CUR, which are missing one and two methoxy groups in the molecular structure, respectively. DMC and BDMC demonstrate significantly better chemical stability than CUR, and recent studies show that both exhibit anticancer properties. However, the antitumor mechanism of the DMC and BDMC compounds is not fully understood [14,15].

**Figure 1:**
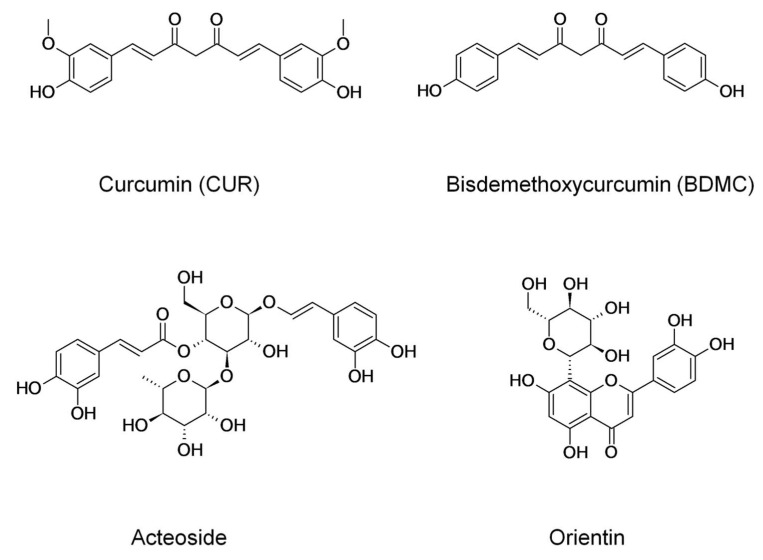
Chemical structures of natural products used in this study: curcumin (CUR); bisdemethoxycurcumin (BDMC); Acteoside, and Orientin.

Acteoside (ACT) belongs to phenylpropanoid glycosides and is formed by making ester and glycosidic bonds, combining caffeic acid, hydroxytyrosol, glucose and rhamnose [16]. It reveals anti-inflammatory, analgesic, immunosuppressive, immunomodulating, anticancer, antimicrobial, and hepatoprotective properties [17]. The efficacy of the antitumor activity of ACT has been observed so far in the treatment of melanoma [18], colorectal [19], and prostate cancers [20]. Orientin (ORI) is a C-glycoside flavonoid equipped with four phenolic, one ketone and two ether groups. It has been extracted from medicinal species of plants such as *Ocimum sanctum*, *Jatropha gossypifolia*, *Phyllostachys*, *Passiflora* and *Trollius* species. ORI reveals many biological activities such as antioxidant, antiviral, antibacterial, anti-inflammation, antidepressant-like, anti-aging, and anticancer effects [21,22]. Chemical formulas of the chemical compounds are shown in Figure 1.

**Figure 2:**
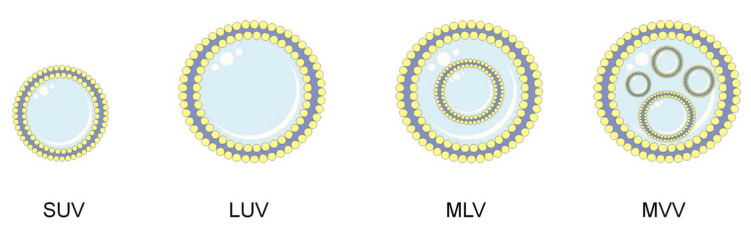
Types of liposomes: SUV—small unilamellar vesicles, LUV—large unilamellar vesicles, MLV—multilamellar large vesicles, MVV—multilamellar large vesicles.

One of the approaches to improve the delivery of natural substances is to use drug carriers, which provide protection to the carried substances as well as offering the potential of an increased concentration of the drug in the target site. One of the more widely studied and broadly used drug delivery systems are liposomes, which are spherical structures whose wall is formed by a double layer of amphiphilic phospholipids enclosing an aqueous core. The hydrophilic parts of the lipids are oriented towards the water phase, while the lipophilic non-polar chains of fatty acids occupy a position opposite each other, creating an inner hydrophobic layer (Figure 2). Liposomes have the ability to encapsulate both hydrophilic and hydrophobic drugs. Hydrophilic drugs are located in their inner aqueous core, while lipophilic drugs are placed in the phospholipid bilayer. Liposomes are non-toxic, biocompatible, and biodegradable and therefore have found application as a drug delivery system. They increase the therapeutic efficacy of drugs by improving chemical and biological stability, increasing biodistribution, and reducing the compounds’ systemic toxicity [23,24,25]. The classification of liposomes, including their size and the number of layers, is presented in Figure 2. According to the literature data, small unilamellar vesicles (SUV) have a diameter within a range of 50–100 nm, where a diameter above 100 nm is classified as large unilamellar vesicles (LUV). Multilamellar large vesicles (MLV) include liposomes of 100–1000 nm in size, the envelope of which is made of multiple lipid bilayers, whereas multivesicular vesicles (MVV) are formed as a by-product during the production of MLV [25,26,27].

SUV, LUV and MLV liposomes are good candidates for drugs with versatile delivery, including oral administration, while MVV liposomes can only be used for parenteral delivery [27]. The small size of the SUV promotes the distribution of medicinal substances in the body and protects against SUV uptake after administration by the mononuclear phagocyte system (MPS) in the liver and spleen. Following intravenous administration, LUVs are rapidly taken up by the MPS system [28]. The rate at which liposomal drugs are cleared from circulation depends on the rate and extent of both drug release and uptake of liposomes through MPS [29]. It has been proven that liposomes with a size of 100–200 nm are retained in the blood without degradation. Extending the liposome’s lifetime despite multiple modifications is a continuing challenge [30]. The delivery of hydrophilic drugs can be improved with the use of unilamellar LUV liposomes by taking advantage of their larger interior surface. What is more, the sensitivity of macrophages to the charge and size of the liposomes can be exploited, and thus the uptake of liposomes by these phagocytic cells can be regulated [31]. It is worthwhile to mention that SUV liposomes diffuse rapidly into the lymphatic capillaries when administered subcutaneously, while larger liposomes remain at the injection site longer. In addition, local lymph nodes retain large liposomes more effectively than SUV liposomes [32].

Herein, we present the research on the possibility of encapsulating selected compounds (CUR, BDMC, ACT, ORI) in liposomal carriers for drug delivery and combining substances to provide an advantage in the anticancer potential towards GBM cell lines. Moreover, we demonstrate the methodology of preparing liposomal formulation composed of two types of lipids by co-embedding more than one substance, thus obtaining synergy in anticancer activities. In addition, an alternative method of liposome extrusion is demonstrated. In order to determine the physicochemical properties of the new drug carriers, NMR and EPR studies were carried out.

## 2. Materials and Methods

### 2.1. Chemical Compounds and Reagents

Curcumin (CUR), (1E,6E)-1,7-bis(4-hydroxy-3-methoxyphenyl)hepta-1,6-diene-3,5-dione, was obtained from Fluorochem (Derbyshire, UK). Bisdemethoxycurcumin (BDMC), (1E,6E)-1,7-bis(4-hydroxyphenyl)hepta-1,6-diene-3,5-dione, was prepared according to a literature procedure [33].

Acteoside (ACT), (2R,3R,4R,5R,6R)-6-[2-(3,4-dihydroxyphenyl)ethoxy]-5-hydroxy-2-(hydroxymethyl)-4-{[(2S,3R,4R,5R,6S)-3,4,5-trihydroxy-6-methyloxan-2-yl]oxy}oxan-3-yl-(2E)-3-(3,4-dihydroxymethyl)prop-2-enoate, was isolated from *Plantago lanceolata* L. as described previously [34].

Orientin (ORI), 2-(3,4-dihydroxyphenyl)-5,7-dihydroxy-8-[(2S,3R,4R,5S,6R)-3,4,5-trihydroxy-6-(hydroxymethyl)oxan-2-yl]chromen-4-one, was isolated from *Adonis vernalis* L. as described earlier [35].

1-Palmitoyl-2-oleoyl-glycero-3-phosphocholine (POPC) and 1,2-dioleoyl-3-trimethylammonium-propane (DOTAP) were obtained from Avanti Polar Lipids (Birmingham, AL, USA). Chloroform, methanol, and dimethylsulfoxide for the preparation of stock solutions for the liposomes’ preparation were obtained from Sigma-Aldrich (St. Louis, MO, USA). Dulbecco phosphate-buffered saline (PBS) was purchased from Sigma-Aldrich.

HPLC grade acetonitrile, water, acetic acid, and potassium chloride were obtained from Avantor Performance Materials (Gliwice, Poland).

Reagents used for in vitro experiments such as fetal bovine serum (FBS), phosphate-buffered saline (PBS), trypsin-EDTA, L-glutamine, dimethylsulfoxide (DMSO), 3-(4,5-dimethylthiazol-2-yl)-2,5-diphenyltetrazolium bromide (MTT), were obtained from Sigma Aldrich.

### 2.2. Liposome Preparation

**Figure 3:**
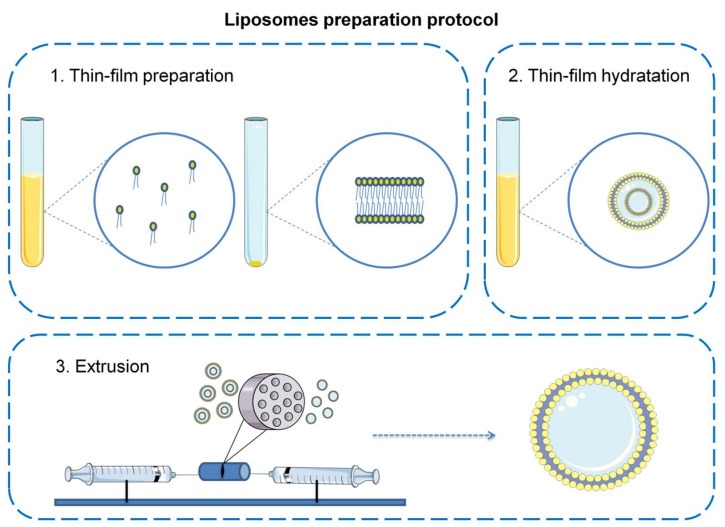
Schematic representation of liposomes preparation protocol.

Briefly, compounds and lipids were dissolved in chloroform (CUR), methanol (BDMC, ACT), and DMSO (ORI). The solvents from appropriate compounds and lipid mixtures were evaporated under reduced pressure using a combination of two methods: high-vacuum rotary evaporation in 30 min and drying under a vacuum pump in 24 h to form a dry lipid film, rehydrated in PBS, and the Avanti^®^ Polar Lipids Mini Extruder kit (Merck KGaA, Darmstadt, Germany) was used to obtain an SUV/LUV. A polycarbonate membrane with a pore size of 100 nm was used (Figure 3). In the applied alternative extrusion method, an ultrasonic UV 2070 homogenizer with an MS 73 head (BANDELIN electronic GmbH & Co., Berlin, Germany) was used.

Liposomes were prepared according to a literature procedure [36] from 1-palmitoyl-2-oleoyl-sn-glycero-3-phosphocholine (POPC) and 1,2-dioleoyl-3-trimethylammonium-propane (DOTAP) mixture (8:2), and either CUR, BDMC, ORI, or ACT or their combination with the following molar ratio of 0.1:8:2 (CUR/ORI/ACT/BDMC:POPC:DOTAP), 0.05:0.05:8:2 (CUR:ORI:POPC:DOTAP), and 0.05:0.05:8:2 (ACT:BDMC:POPC:DOTAP). The final concentrations were as follows: 36.8 µg/mL CUR, 130.8 µg/mL BDMC, 44.8 µg/mL ORI, 62.5 µg/mL ACT, 6 080.6 µg/mL POPC, 1 397.1 µg/mL DOTAP and mixtures of compounds, respectively, 18.4 + 22.4 µg/mL CUR + ORI and 15.4 + 31.2 µg/mL BDMC + ACT.

### 2.3. Liposome Size, Polydispersity Index, and Zeta Potential Measurement

Liposome size, Polydispersity Index (PDI), and Zeta potential were measured using Zetasizer Nano ZS (Malvern Instruments, Malvern, UK). This system is based on dynamic light scattering (DLS technology, Ottawa, Canada). Measurements were performed in triplicate. To achieve optimal concentration of particles, 10 µL of liposome suspension was diluted with 10 mL of water. U-shaped cuvettes with a gold electrode were used.

### 2.4. HPLC Analysis and Encapsulation Efficiency

The concentration determination was performed using the HPLC method. The developed method was carried out in gradient conditions with UV-VIS detection on analytical apparatus comprising a 1220 Infinity LC chromatography system (Agilent Technologies, Santa Clara, CA, USA) equipped with a DAD detector, a G1315C optical unit, a column oven, and an autosampler. The gradient elution was based on the mobile phase consisting of two solvents: A—acetonitrile for HPLC, and solvent B—acetic acid in water (1 mL CH_3_COOH in 1000 mL of water) with a constant flow rate of 1 mL/min during the whole run time (25 min). The volume ratio (*v/v* in %) of the mobile phase (A:B) started from 15/85 at 0 min, changed to 25/75 at 10 min, later changed to 70/30 at 18 min, and returned to the initial ratio of 15/85 at 25 min. The stationary phase was octadecylsilyl silica gel for chromatography (Lichrospher^®^ 100 RP-18 column, 25 × 4 mm; 5 µm, Merck KGaA), stored at 25.0 ± 1.0 °C. The injection volume was 60 μL. The gradient program was allowed to separate all investigated analytes (CUR, BDMC, ORI, ACT), which were identified by specific wavelength, i.e., 270 nm for ORI, 330 nm for ACT and 420 nm for BDMC and CUR. The retention time for ORI was 9.5 min, ACT was 12.9 min, for BDMC was 20.4 min, and for CUR 20.9 min. Each sample was injected in triplicate. The validation of the HPLC method concerning selectivity, precision, linearity, range, and limits of detection and quantitation was performed according to the ICH guidelines for analytical method validation (Q2(R1)) [37].

The encapsulation efficiency (EE) of CUR, BDMC, ORI, ACT and their mixtures was calculated according to the formula [38]:

EE = C_en_/C_in_ × 100% (1)

where C_en_—is the actual amount of the substance in the liposomes measured after their disruption using HPLC, C_in_—is the initial amount of the substance used for the preparation of the liposomes.

### 2.5. NMR and EPR Measurements

The ^1^H NMR experiments were performed using a homemade NMR pulse spectrometer, which operates at 30.2 MHz. All measurements were carried out under static conditions at room temperature. 

The proton spin–lattice relaxation time *T_1_* was obtained using a saturation pulse sequence [n* π/2 − t − π/2]. The series of RF π/2 pulses at an interval of several hundred μs caused the saturation of the magnetization. Then, after time t the second π/2 pulse was applied, and the free induction decay FID was observed. For all samples, recovery of magnetization was one-exponential. The relaxation time *T_1_* was determined by fitting the following equation to the experimental data:

(2) $$M\left(t\right)=M_{0}\left(1-exp\left(-\frac{t}{T_{1}}\right)\right)$$

where *M_0_* is the equilibrium magnetization. The measurement error was 3%. A detailed description of the apparatus was published elsewhere [39,40,41].

EPR measurements were made on the Adani X-band continuous wave spectrometer. The tested substances with a volume of 15 µL were closed in a special thin-walled 50 µL capillary, which was placed in the resonator looking for the minimum electric field for maximum sensitivity. Earlier, for reference, the maximum sensitivity of the apparatus was determined for the same volume of the aqueous 3CPy spin probe solution, which is 500 pM. The 3CPy spin probe is characterized by three lines separated by approximately 32 G. A wide scan range was selected for testing.

Spectrometer parameters: second modulation frequency 10 kHz, second modulation amplitude 150 μT, RF output power 9 mW, center field 336.9 mT, sweep time 120 s number of scans 2, sweep width 200 mT, resonator type: cavity.

The radical scavenging measurements were performed on a spectrometer operating at a resonance frequency of 1150 MHz, equipped with a surface resonator manufactured by Novilet.

The measurement was performed in the same routine at a room temperature of 25 °C. To the aqueous solution of the 3CPy spin probe with a volume of 60 µL and a concentration of 100 µM, 20 µL of the test substance was carefully added by the pipette. After adding the substance, the whole sample was not mixed mechanically, only diffusion processes took place. Spectra were recorded every 3 s for over 1000 s each. The ratio of the volume of the spin probe to the test substance was chosen in a way that minimized the decay of the EPR signal of the spin probe due to dilution. Standard microtubes with cap type Vitraton Akes 01298-00 with a volume of 500 μL, which were placed in a loop resonator, were used for the measurements.

Spectrometer parameters: second modulation frequency: 20 kHz, second modulation amplitude: 1 G, RF output power: 47 mW, resonance centre frequency: 1157 MHz, sweep time: 3 s, sweep amplitude: 50 G, resonator type: surface LGR.

### 2.6. Biological Activity Assessment (MTT Test)

T98G and U-138 MG GBMcells were provided by the European Collection of Authenticated Cell Cultures (ECACC, Salisbury, UK), and American Type Culture Collection (ATCC, Manassas, VA, USA), respectively. Non-cancerous MRC-5 were also obtained from ATCC (Manassas, VA, USA). GBM cells were maintained in ATCC-formulated Eagle’s Minimum Essential Medium (Merck KGaA, Darmstadt, Germany) and normal cells were cultured in Dulbecco’s Modified Eagle’s Medium (Merck KGaA, Darmstadt, Germany), containing 10% fetal bovine serum (EURx, Gdańsk, Poland) and 1% of antibiotic solution (Sigma-Aldrich, St. Louis, MO, USA) at 37 °C, in a humidified, 5% CO_2_ atmosphere. To assess the effect of the compounds and their formulations on GBM and normal MRC-5 cells viability, 1 × 10^4^ cells/well were seeded on 96-well plates. After 24 h of the initial incubation, the cells were treated with compounds or their formulations in the concentrations of 1–100 µM and 1–50 µM, respectively. Cells treated with 0.1% of dimethylsulfoxide (DMSO) and empty liposomes DOTAP:POPC were used as two separate controls. Incubation lasted for 24 h, and the cells were then harvested.

The effect of the tested compounds on cell viability was assessed by the MTT assay, following the standard protocol described elsewhere [42]. Briefly, after 24 h of incubation with the analyzed compounds or their mixtures, the cells were washed twice with phosphate-buffered saline (PBS) and further incubated for 4 h with a medium containing 0.5 mg/mL 3-(4,5-dimethylthiazol-2-yl)-2,5-diphenyl-2*H*-tetrazolium bromide (MTT). Then, the formazan crystals were dissolved in acidified isopropanol, and the absorbance was measured at 570 nm and 690 nm. All experiments were repeated three times.

### 2.7. Western Blot Analysis 

Whole cell lysates were prepared from U-138 MG and TG98 cells using the standard radioimmunoprecipitation assay (RIPA) buffer and protease inhibitor (Merck KGaA or Sigma-Aldrich).

Lysates were separated on 12% (caspase-3) or 10% (p53) SDS-PAGE slab gels. Proteins were transferred to the nitrocellulose Immobilon P membrane. After blocking for 2 h with 10% skimmed milk, proteins were probed with mouse anti-p53, and mouse anti-caspase-3 antibodies (Santa Cruz Biotechnology, Dallas, TX, USA). Alkaline phosphatase AP-labeled anti-mouse IgG secondary antibodies (BioRad Laboratories, Hercules, CA, USA) were used in the staining reaction. Bands were visualized using the AP Conjugate Substrate Kit NBT/BCIP. The amount of immunoreactive products in each lane was determined using the ChemiDoc Imaging System (BioRad Laboratories). Values were calculated as relative absorbance units (RQ) per mg of protein and expressed as a percentage of the control.

### 2.8. Statistical Analysis

GraphPad Prism 9 (GraphPad Software, San Diego, CA, USA) was used to analyze the data. To assess the significance of the differences in the evaluated parameters one-way ANOVA with Dunnett’s post-hoc test was performed with the significance level of *p* < 0.05.

## 3. Results and Discussion

### 3.1. Liposome Size, Encapsulation, Polydispersity Index, and Zeta Potential Measurement

A diverse MLV population was obtained by the method of hydration of a thin lipid film, which in detail relies firstly on the preparation of a dry lipid film and secondly on its hydration by an aqueous solution during vigorous shaking and ultrasonic bath treatment. Next, the extrusion method was used to obtain a homogeneous population of liposomes (SUV/LUV). 

**Figure 4:**
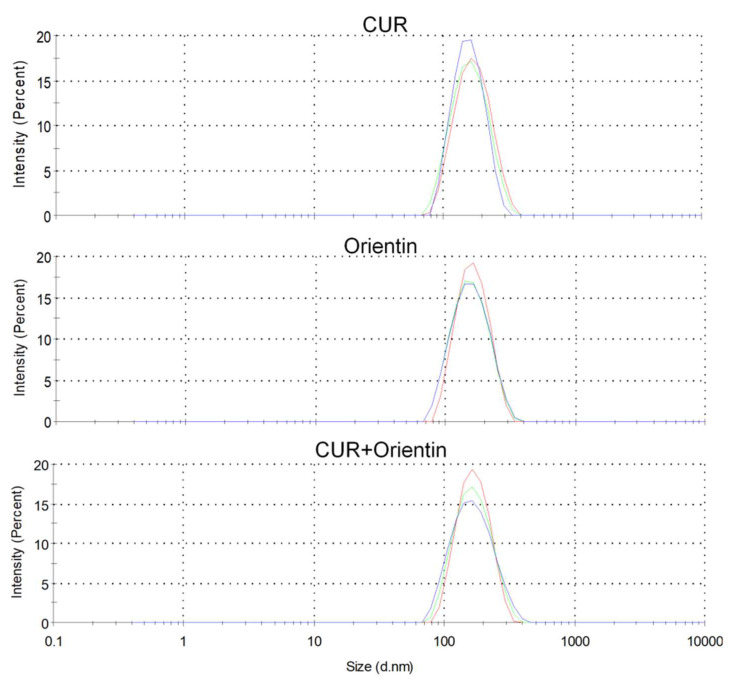
The size of the liposomes (with CUR, ORI, and mixture of CUR + ORI) was measured with dynamic light scattering (DLS).

The liposomes were evaluated for their particle size, including PDI and Zeta potential, to indicate the homogeneity and stability of the nanoformulation. The size of the liposomes was measured in triplicate immediately before the MTT test. The obtained reproducibility of the results is necessary for further interpretation of in vitro tests. The results are presented in Figure 4 (for CUR, ORI, and mixture of CUR and ORI) and Table 1. The average particle size of the prepared liposomes was below 190 nm with a narrow size distribution, which confirms a homogeneous distribution. Such adequate uniformity is necessary to increase the biodistribution potential in subsequent in vivo activity studies. The size of the nanocarriers is crucial for cellular uptake. Nanoformulations up to 300 nm reveal good penetration through the membrane in the process of macropinocytosis [43]. Briefly, particle size analyses of liposomal preparations were performed using dynamic light scattering (DLS), whereas the zeta potential was determined using electrophoretic light scattering (ELS) available on the Zetasizer Nano ZS.

The electric double layer around each nanoparticle consists of an inner Stern layer and a so-called outer diffusion layer. The potential at this boundary is called the Zeta Potential. It provides information on the nature and functionality of the nanoliposome surface and is an important parameter characterizing the drug delivery system [44,45]. If all particles have a significant negative or positive Zeta Potential (<−30 mV or >+30 mV), they repel each other. Hence it may be concluded that the system is very stable [46]. In the herein presented study, zeta potential values ranged from +37.0 to +38.8 mV, which confirms the cationic character of obtained liposomes and the stability of the particle suspension [45].

In order to achieve the desired antitumor effect, the following concentrations of compounds in lipids were used: 36.8 µg/mL CUR, 130.8 µg/mL BDMC, 44.8 µg/mL ORI, 62.5 µg/mL ACT, 6080.6 µg/mL POPC, 1397.1 µg/mL DOTAP and mixtures of compounds, respectively, 18.4 + 22.4 µg/m CUR + ORI and 15.4 + 31.2 µg/mL BDMC + ACT.

The encapsulation efficiency for the above concentrations was: 83.1 ± 3.7% for CUR, 79.3 ± 1.4% for ORI, and 89.9 ± 0.9% for CUR from the mixture (CUR + ORI) and 92.2 ± 2.0% of ORI from the mixture (CUR + ORI), and accordingly 80.8 ± 2.9% for BDMC, 99.2 ± 1.8% for ACT, and 87.3 ± 3.1% for BDMC from the mixture (BDMC + ACT) and 82.5 ± 3.9% of ACT from the mixture (BDMC + ACT).

### 3.2. NMR Measurements

NMR measurements provide valuable information about the molecular relaxation and line shapes in liposome systems, where molecules form aggregates and reorient with respect to the external magnetic field. The molecular motion in such systems is mainly due to the tumbling of aggregates or diffusion [47]. Proton NMR line shapes are determined by dipolar interactions modulated by internal motions [48]. When the modulation is fast, the line shape is Lorentzian. This is called the motionally narrowed limit and is commonly observed in solution when NMR is performed. However, for slow anisotropic motions, the line shape is Gaussian [49]. The linewidth (Δν_1/2_) at half-height of the NMR signal is related to spin–spin relaxation time *T*_2_, which describes the rate of the decay of the transverse magnetization:

(3) T2~1Δ1/2

In different liposome systems, both components related to the solid and liquid phases can contribute to the line shape, and therefore their determination and separation are essential for interpreting the NMR spectra.

**Figure 5:**
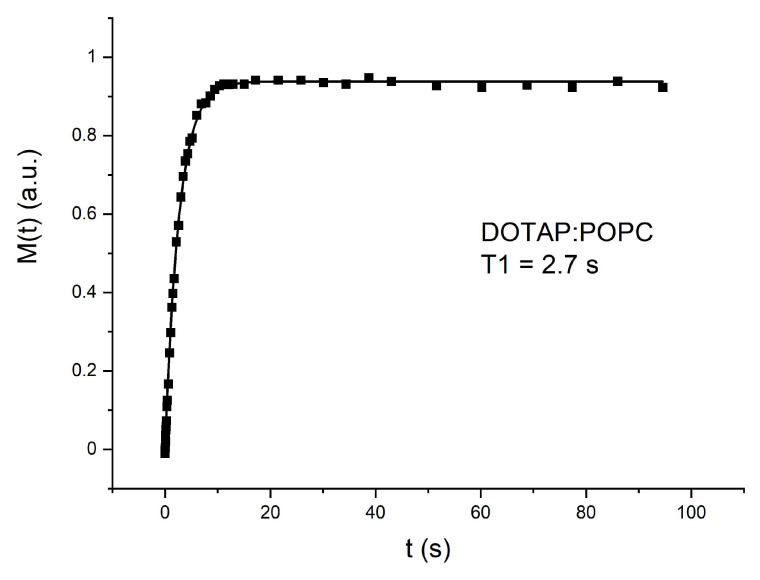
An example recovery of the magnetization as a function of time t for empty liposomes DOTAP:POPC.

An example of magnetization recovery is shown in Figure 5.

**Figure 6:**
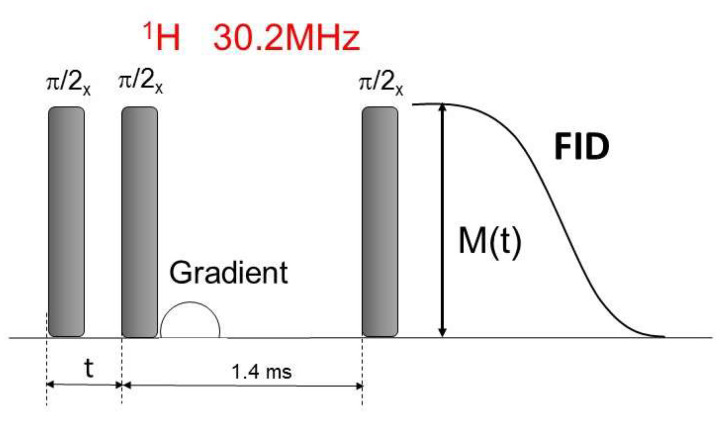
The pulse sequence for the measurement of the spin–spin relaxation time *T_2_*_._

The spin–spin relaxation time *T*_2_ in the laboratory frame was determined using a pulse sequence presented in Figure 6.

After the first $\left(\pi /2\right){/}_{x}$ pulse at a variable time t the second $\left(\pi /2\right){/}_{x}$ pulse was applied. Then the gradient *Gr* was used to accelerate the decay of transverse magnetization components. The third $\left(\pi /2\right){/}_{x}\;$ pulse was applied after the time of about 1.4 ms, and the FID signal was observed.

**Figure 7:**
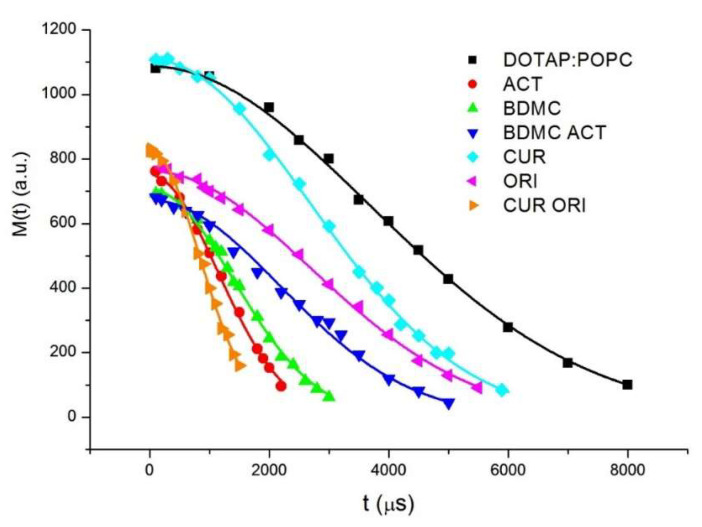
The decay of the magnetization obtained in the experiment for the measurement of the spin–spin relaxation time T_2_ for all liposome samples.

Figure 7 presents the FID signals obtained in the experiment for the measurement of the spin–spin relaxation time *T_2_*. For all T_2_ measurements, the decay of magnetization was one-exponential and described by the Gaussian function:

(4) $$M\left(t\right)=M_{0}exp\left(-{\left(\frac{t}{T_{2}}\;\right)}^{2}\right)$$

The values of the spin–lattice *T_1_* and spin–spin *T_2_* relaxation times obtained for all samples are collected in Table 2.

The obtained results indicate that for all samples, the values of the relaxation time *T_1_* are the same within the error limits. However, the spin–spin relaxation times *T_2_* are different for empty liposome system DOTAP:POPC and liposomes with compounds incorporated. It can be assumed that the spin–lattice relaxation time *T*_1_ is dominated by molecular and intermolecular motions, which is characteristic of a mobile fluid. On the other hand, the spin–spin relaxation time *T_2_* depends on the orientation rate of molecules, and according to Equation (3) it is inversely proportional to the linewidth. The longest T2 time for a pure liposome system indicates that isotropic motion such as tumbling of liposomes and lateral diffusion is averaged and gives narrow absorption lines in the NMR spectra [50]. The significantly shorter *T_2_* relaxation times for liposome systems with the embedded substances indicate the broadening of the line caused by presumably the restricted anisotropic motion.

### 3.3. EPR Measurements

#### 3.3.1. X Band Measurement for the Presence of Stable Free Radicals

**Figure 8:**
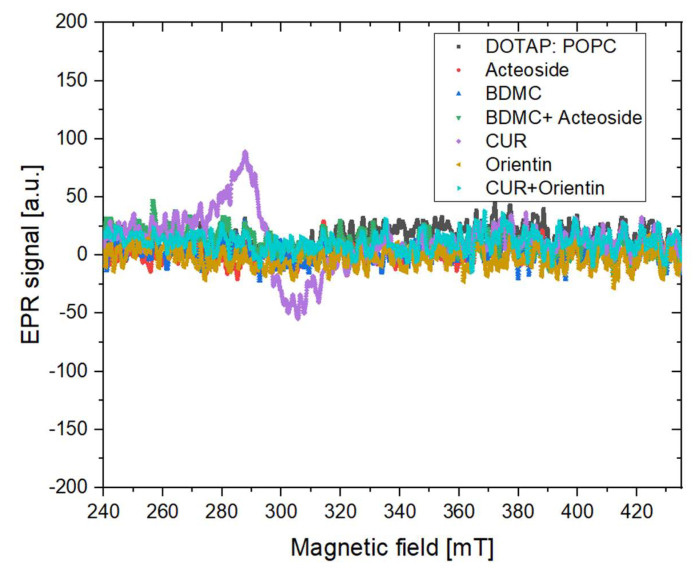
X band measurement results performed to investigate the presence of the stable free radicals in the liposome samples; CUR—curcumin, BDMC—bisdemethoxycurcumin.

The results obtained in the study are summarized in Figure 8. No paramagnetic centers were observed for any tested substances apart from the CUR sample, which complies with the literature data [51].

For CUR sample, the EPR line parameters were calculated: line width 17.42 mT, g-factor 2.29, amplitude pp 125 [a.u.].

#### 3.3.2. L Band Free Radical Scavenging

The use of a spectrometer operating at a relatively low frequency gives possibilities to measure easy and relatively fast high volumes (more than 10 µL) of liquid samples. There is no limitation related to the difficulty of tuning the system and the maximum volume of liquid in the resonator, as is the case with X band spectrometers. Moreover, the test substance can be added in real-time without causing technical problems related to system detuning and the inability to perform correct measurements. The dynamic measurements can be performed and acquire spectra even every 1 ms, if necessary the in vivo experiment as described in [52]. Moreover, such a solution enables the analysis of the over-modulated signal with the use of multiharmonic analysis, which additionally improves the S/N ratio [53,54]. In addition, chemical reactions take place better in larger volumes under more realistic conditions.

Each time a spectrum covering three lines of a stable spin probe was recorded. Thanks to this, it is possible to analyze the data in a more favorable way by averaging the results collected for each of the three lines for one scan. For the proper analysis, the collected data were normalized.

**Figure 9:**
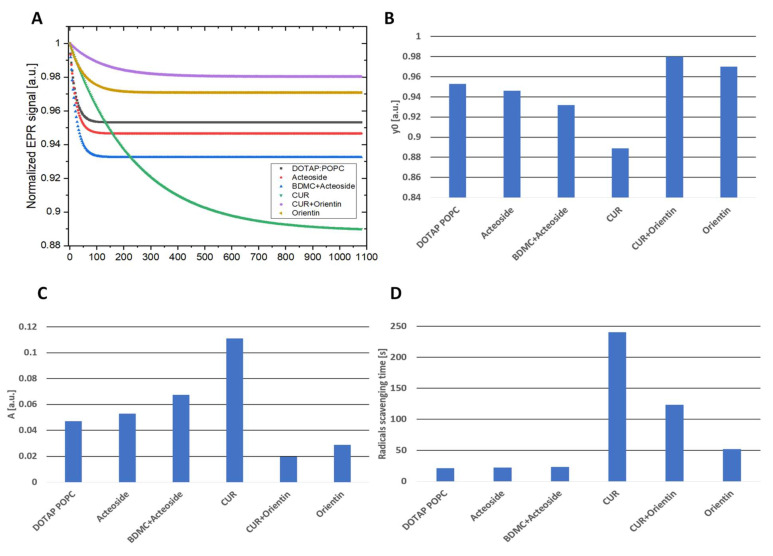
(**A**) L band radical scavenging results. (**B**) The number of remaining radicals of the 3CPy spin probe after mixing with the liposomal formulations (in arbitrary units). (**C**) The amount of the swept-off radicals of the 3CPy spin probe after mixing with the liposomal formulations (in arbitrary units). (**D**) The scavenging reaction time in seconds; CUR—curcumin, BDMC—bisdemethoxycurcumin.

Exponential fits (y_0_ + A × exp (−x/T)) were made to the experimental results to determine the decay T time constant reaction, y_0_—gives information on how many radicals are left and parameter A—informs in arbitrary units how many radicals have been swept away. The results are summarized in Table 3 and Figure 9.

It was observed that pure CUR allows for radical scavenging, and the reaction takes place under the described conditions for even about 240 s. CUR added to the active substances of ORI slows down the radical scavenging reaction and reduces the possibility of recombination of the radicals originating from the spin probe, leading to a deterioration of their removal capacity by about thirty percent. The scavenging reaction time for DOTAP:POPC, ACT and BDMC + ACT samples is comparable, while the most efficient in terms of the number of radicals removed is BDMC + ACT.

### 3.4. Biological Activity

Recent research shows that scientific advances, including new therapeutic substances, their combinations, and modern treatment methods, are contributing to the improvement of the quality and life expectancy of cancer patients [55]. Unfortunately, the initial positive response to the chosen treatment is often insufficient and leads to tumor recurrence. The problem is the development of multidrug resistance (it is responsible for 90% of chemotherapy therapeutic failures), including resistance also to new drugs [56]. Multiple drug resistance (MDR) is defined as resistance to one group of anticancer drugs and the related resistance that also occurs to other medications, the structures, and mechanisms of action could be completely different. MDR may occur through a variety of mechanisms. Drug outflow is mainly observed, primarily influenced by overexpression of ABC transporters in the membranes of cancer cells. Other mechanisms include inhibition of apoptosis, alteration of drug metabolism, genetic factors, reduction in drug penetration, and downregulation of binding to cancer cells by altering the expression level of drug binding proteins. Therefore, new therapeutic approaches are constantly being sought. This manuscript presents a selection of natural phytochemicals (CUR, BDMC, ACT, and ORI) with promising anticancer potentials against GBM cell lines. Yan Liang et al. indicated that natural products like curcuminoids present certain advantages [57]. They are succored antitumor drugs with or without nanocarriers that maximize the synergistic effects against MDR in tumor cells [57,58,59]. Tseng et al. reported that CUR and tetrahydrocurcumin are effective substances for the treatment of acute myeloid leukemia with MDR (Ara-C resistance) through the mechanisms of apoptosis and autophagy [60]. In another study, BDMC was used to reverse MDR in cisplatin-resistant non-small cell lung cancer by inhibition of CA916798 mRNA and protein and PI3K/AKT activities [61]. Despite advances and new strategies for treating GBM, MDR is a significant cause of failure for commonly used chemotherapeutic agents, including temozolomide (TMZ) [62,63]. Other causes include innate tumor cell resistance, inadequate drug concentration due to the blood–brain–tumor barrier, molecular heterogeneity, and clinical trials of new drugs in small groups [64].

In this study, empty liposomes were first tested to select a non-toxic lipid composition, while at the same time obtaining a cationic charge that would allow compounds encased in nanocarriers to cross the blood–brain barrier. Liposomes composed of POPC and DOTAP:POPC lipids in the 1:9 and 2:8 ratio were tested. All nanoformulations did not reduce cell viability, regardless of the lipids used and the ratio of DOTAP:POPC. Thus, only the DOTAP:POPC formulation was selected in a ratio of 2:8 to obtain the most cationic liposomes and used for further research. Firstly, the cytotoxic activity of the tested compounds in their free form was determined after 24 h, and the inhibitory concentration (IC_50_) was calculated, as shown in Table 4. 

Interestingly, Tae Woong Hwang et al. reported that the combined treatment regimen of ACT with TMZ was effective and showed synergism in action against GBM [65]. The main mechanisms involved in apoptosis and autophagy were the increase in level cleavage caspase-3 and phosphorylated p53 and decreased Bcl-2. These studies confirm the significant potential of ACT in the treatment of GBM [65]. Moreover, in other studies Wei-Qiang Jia et al. observed the effect of ACT on the reduction in GBM cell viability and activation of autophagy in cells. These effects were possible by increased let-7g-5p and decreased HMGA2 through the blockade of Wnt/β-catenin signaling pathway [66].

In this study, we combined CUR with ORI and BDMC with ACT because these separate compounds showed the highest cytotoxicity against selected GBMcell lines. Moreover, these compounds probably reveal different anticancer mechanisms and can modulate the signaling pathways in other parts or various places [67,68,69].

**Figure 10:**
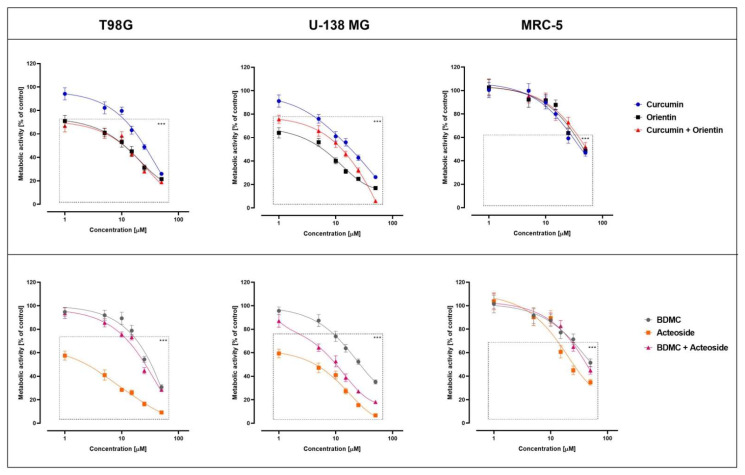
The cytotoxic activity of liposomal formulation containing CUR, BDMC, ACT, ORI and their combination against T98G, U-138 MG, and MRC-5 cells. Data are expressed as the mean ± SEM from three independent experiments; BDMC—bisdemethoxycurcumin. Statistical significance was assessed by Tukey’s test (*** *p* < 0.001).

In the next step, the tested compounds were closed in cationic nanoliposomes to improve the bioavailability and biodistribution and to potentially enable their crossing through the blood–brain–tumor barrier [70]. The IC_50_ values of the liposomal formulation of tested compounds and their combination are presented in Table 5, and the dose–response curves are presented in Figure 10.

Kalaiyarasu Thangaraj et al. noted that ORI has shown significant cytotoxicity against HT29 colon cancer cell lines [71]. The cell cycle arrest in the G0/G1 phase, induced intracellular ROS, and the activation of caspase-dependent apoptosis have been described as the main mechanisms of this action. In other studies, Soo-Jin Kim et al. observed that ORI prevents migration and inhibits tumor metastasis or recurrence in MCF-7 breast cancer cells [67]. The indicated studies confirm the use of ORI as a powerful therapeutic compound against cancer cells, which was also confirmed in this study, demonstrating potential against GBL (Table 3). Moreover, natural compound interactions can result in positive effects (synergistic or additive) or adverse effects (antagonistic effects) [72]. In this study, we have confirmed the synergistic effect of the CUR + ORI mixture in liposomes. Despite using half the final concentrations for the combination (18.4 + 22.4 µg/mL CUR + ORI, 50 µM + 50 µM), it showed a similar cytotoxic effect to free ORI (44.8 µg/mL) (Figure 10). Therefore, it is proposed that the combination of these phytochemicals could induce a beneficial therapeutic outcome against GBL. Moreover, the use of liposomal nanoformulation increased the cytotoxicity of this combination. Aditi Jhaveri et al. made similar conclusions by encapsulating resveratrol in liposomes [73]. This increased the compound’s activity in vitro and contributed to a favorable response in vivo compared to the free compound. In another study, A. Majchrzak-Celińska et al. observed that the combination treatment of curcuminoids and sodium butyrate can also reveal a promising effect against GBM [74].

**Figure 11:**
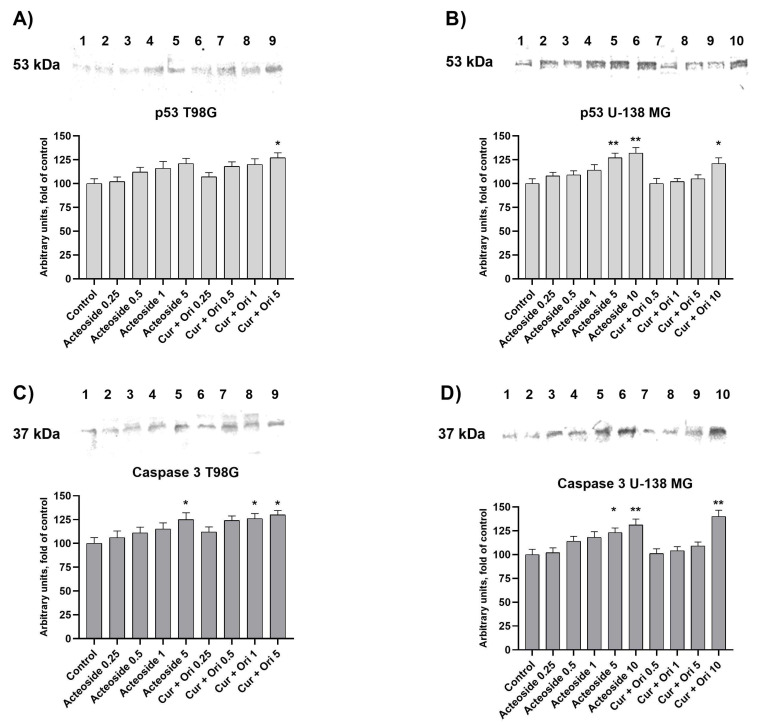
The effect of ACT and CUR+ORI on the protein level of p53 in panels (**A**) and (**B**) and caspase-3 in panels (**C**) and (**D**) in GBM cells. Data are presented as mean ± SEM. * represents *p* < 0.05, ** represents *p* < 0.01, compared with the untreated group. The values in the x axes are concentration in µM of ACT or the 1:1 molar mixture of CUR + ORI. The representative immune blot respectively for Figure 11 show in Figures S1–S4.

In order to determine the mechanism of action of the studied selected liposomal formulations, we analyzed the level of p53 and Caspase-3 proteins expression in both tested GBM cell lines (Figure 11).

Liposome-entrapped ACT (0.25–5 μM), and CUR + ORI (0.25–10μM) increased the protein level of p53 and caspase-3, the key apoptotic effectors, in a dose-dependent manner. Compared with the control cells, ACT and CUR + ORI at their IC_50_ concentrations increased p53 in T98G and by 21% and 27%, respectively, and in U-138 MG by 32% and 21%, respectively. The level of caspase-3 expression increased by 25% and 30% due to the presence of ACT and CUR + ORI at their IC_50_ concentrations, respectively, while in U-138 MG by 31% and 40%, respectively. The most significant inducer of caspase-3 was CUR + ORI at the dose of 10 µM (5 + 5 µM; 1.84 + 2.24 µg/mL) in U-138 MG cells. In turn, in the T98G cell line we observed a significant increase under the influence of ACT at the dose of 5 µM (3.125 µg/mL) and CUR + ORI at the doses of 5 µM (2.5 + 2.5 µM; 0.92 + 1.12 µg/mL) and 10 µM (5 + 5 µM; 1.84 + 2.24 µg/mL).

Fang An et al. reported that ORI and vitexin have an anticancer effect on EC-109 esophageal cancer cells, which was associated with the regulation of p53 and Bcl-2 gene expression [75]. However, the concentration-dependent increase in p53 protein was not as significant as in our study. In another study, the increase in p53 protein was noted after ORI treatment on colorectal carcinoma HT29 cells [71]. Moreover, an increase in caspase-3 has not been previously observed for the antitumor effect of ORI and its mixtures.

Interestingly, Tae Woong Hwang et al. proved that the combination treatment of ACT and TMZ showed synergism in effect on GBM cells by increasing the expression of proteins associated with apoptosis. However, the impact of ACT alone on p53 protein levels and caspase 3 was not as significant as in our study, which confirms the importance of using nanoformulations [65].

These presented results confirm the possibility of using DOTAP:POPC liposomes to encapsulate selected compounds. There is a significant increase in the cytotoxicity of the compounds in the liposomes as compared to the free compounds. These conclusions confirm the liposomal preparation’s advantage in the context of better effectiveness of natural substances against T98 and U-138 MG GBM cell lines. Moreover, the entrapment of compounds in positively charged liposomes increases the passage of these compounds through the blood–brain barrier.

### 3.5. The Alternative Method of Liposome Extrusion

**Figure 12:**
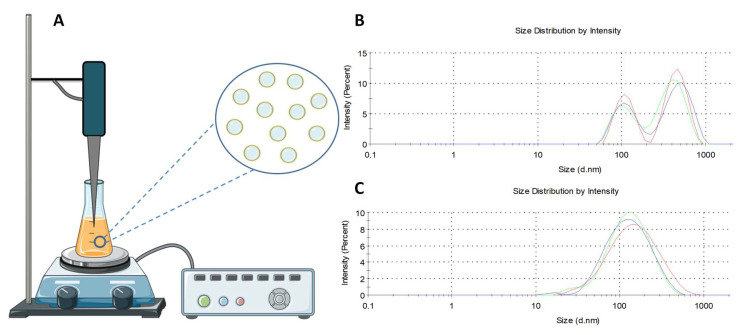
Schematic representation of set for the alternative method of liposome extrusion (**A**). The size of the liposomes with curcumin was measured with dynamic light scattering (DLS)—hydrated and untreated (**B**) and extruded using an ultrasonic UV 2070 homogenizer with an MS 73 head (**C**).

The technique that is most frequently used for preparing liposomes in a small scale is the thin film method. After its preparation, a heterogeneous distribution of liposomes is obtained. The extrusion procedure should then be performed in order to unify their size. The most popular and precise way to do so on a laboratory scale is using an extruder with a polycarbonate membrane with an appropriate pore size [76]. It is a time-consuming process with many disadvantages; however, it provides a formulation uniform in size, which properties are independent of the size distribution as well as repeatable and reproducible. As part of the conducted experiments, we are the first to present a new extrusion method using an ultrasonic UV 2070 homogenizer with an MS 73 head (Figure 12). Nanoformulations were prepared using the thin film hydration method. Then, a UV 2070 homogenizer was used for 1 min with a pulsating work rate (5 s of work, 5 s of rest) at 70% power. This way, liposomes containing CUR were obtained with a homogeneous size distribution of the size below 200 nm (155.5 ± 16.2 nm, 0.26 ± 0.004 PDI, and 37.4 ± 2.0 mV of Zeta Potential), with a broader size distribution compared to a traditional extruder (Avanti^®^ Polar Lipids Mini Extruder kit). The presented method significantly reduces the time related to the extrusion stage. Moreover, this method may enable the scale-up and production of liposomes required for in vivo and clinical research. The described experiments are of a pilot nature and require optimization and further investigation.

## 4. Conclusions

The presented studies provided strong evidence of the possibility of encapsulating four selected natural phytochemicals—CUR, BDMC, ACT, and ORI, as well as their mixtures in cationic liposomal nanoformulation composed of two types of lipids (DOTAP:POPC). The data for the first time indicated that the studied compounds and their combinations encapsulated in liposomes are more cytotoxic against GBM cells (T98G, U-138 MG) in comparison to free compounds. The high cytotoxic effect of liposome-encapsulated ACT on GBM with IC_50_ calculated at 2.9 ± 0.9 µM after 24 h is worth noting. In addition, we have confirmed the synergistic effect of the mixture CUR and ORI in liposomes with IC_50_ calculated at 12.5 ± 2.6 µM after 24 h. Additionally, ACT and the mixture of CUR and ORI were both found to induce cancer cell death by apoptosis, as they increased the expression of p53 and caspase-3. Moreover, we demonstrated an innovative method of liposome extrusion, which allowed us to obtain a homogeneous size distribution below 200 nm. In order to determine the physicochemical properties of the new drug carriers, NMR and EPR studies were carried out. This methodology could be considered as a promising tool allowing an increase in the concentration of natural compounds through their precise delivery into cancer cells, and also to pass the blood–brain barrier.

## Figures and Tables

**Table 1:** The Particle Size, Polydispersity Index (PDI) and Zeta Potential of the liposomes.

Compound	Particle Size (±SD) [nm]	PDI	Zeta Potential [mV]
CUR	168.2 ± 7.5	0.07	+38.8
ORI	163.2 ± 2.6	0.07	+37.0
CUR + ORI	172.5 ± 0.06	0.09	+37.6
BDMC	163.9 ± 5.5	0.07	+37.3
ACT	187.5 ± 3.4	0.11	+37.3
BDMC + ACT	166.7 ± 2.7	0.08	+38.1

CUR—curcumin, BDMC—bisdemethoxycurcumin, ACT—acteoside, ORI—orientin.

**Table 2:** Proton spin–lattice and spin–spin relaxation times for the tested liposomes.

Compound	*T*_1_ (s)	*T*_2_ (ms)
DOTAP:POPC	2.7	5.3
ACT	2.8	1.6
BDMC	2.6	2.0
BDMC + ACT	2.7	3.0
CUR	2.7	3.7
ORI	2.6	3.8
CUR + ORI	2.3	1.2

CUR—curcumin, BDMC—bisdemethoxycurcumin, ACT—acteoside, ORI—orientin.

**Table 3:** Parameters determined in the L band EPR measurements for the liposomal formulations.

Parameters	DOTAP:POPC	ACT	BDMC + ACT	CUR	CUR + ORI	ORI
A [a.u.]	0.047	0.053	0.067	0.111	0.020	0.029
T [s]	21.25	22.2	23.1	240.6	123.29	52.3
y_0_ [a.u.]	0.953	0.946	0.932	0.89	0.98	0.97

CUR—curcumin, BDMC—bisdemethoxycurcumin, ACT—acteoside, ORI—orientin.

**Table 4:** The IC_50_ values of CUR, ORI, BDMC, ACT, and their combinations dissolved in DMSO. Data are expressed as the mean ± SD from three independent experiments.

IC_50_ [µM]		
Compound	Cell Line	
	T98G	U-138 MG
CUR	47.5 ± 3.1	19.0 ± 3.6
ORI	nd	nd
CUR + ORI	nd	24.0 ± 2.3
BDMC	39.0 ± 2.7	40.0 ± 3.9
ACT	85.0 ± 4.3	44.0 ± 4.1
BDMC + ACT	nd	69.0 ± 5.2

nd—the obtained values were higher than 100 µM, CUR—curcumin, BDMC—bisdemethoxycurcumin, ACT—acteoside, ORI—orientin.

**Table 5:** IC_50_ values of CUR, ORI, BDMC, ACT, and their combinations in liposomes. Data are expressed as the mean ± SD from three independent experiments.

IC_50_ [µM]		
Compounds in Liposomes	Cell Line	
	T98G	U-138 MG
CUR	24.0 ± 2.1	19.5 ± 2.4
ORI	12.0 ± 1.7	7.0 ± 1.5
CUR + ORI	12.5 ± 2.6	13.0 ± 1.4
BDMC	29.0 ± 2.2	28.0 ± 1.9
ACT	2.9 ± 0.9	4.0 ± 1.1
BDMC + ACT	23.0 ± 1.8	11.0 ± 1.4

CUR—curcumin, BDMC—bisdemethoxycurcumin, ACT—acteoside, ORI—orientin.

## Data Availability

The data presented in this study are available on request from the corresponding author.

## Supplementary Materials

The following supporting information can be downloaded at: https://www.mdpi.com/article/10.3390/cancers14246222/s1, Figures S1–S4: The representative immune blot respectively for Figure 11.
