# Supplementary material for: Natural Compounds in Liposomal Nanoformulations of Potential Clinical Application in Glioblastoma
Source: Cancers (Basel). 2022 Dec 16;14(24):6222. doi: 10.3390/cancers14246222 (PMC9776450; doi:10.3390/cancers14246222)
Supplement: Supplementary file 1 [file cancers-14-06222-s001.zip › cancers-2098418-supplementary.pdf]

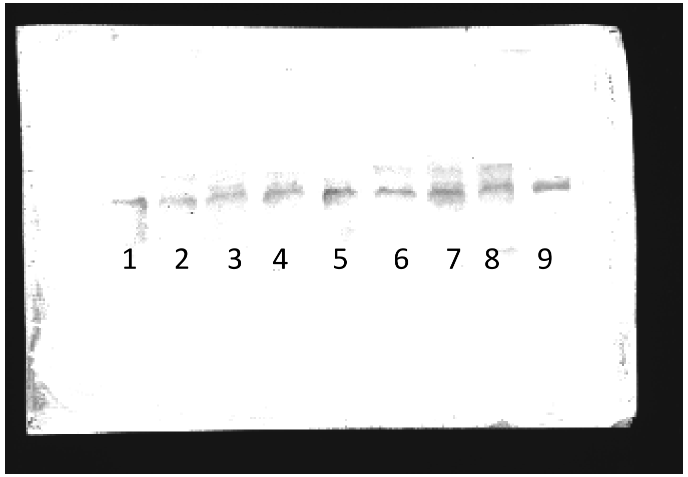

Figure S1. The representative immunoblot respectively for Figure 11.
The level of Caspase -3 protein in T98G cell line.
1 - Control, 2 - Act 0,25 μM, 3 – Act 0,5 μM, 4- Act 1 μM, 5 - Act 5 μM, 6 – CUR+ORI 0,25 μM, 7- CUR+ORI 0,5 μM, 8 - CUR+ORI 1 μM, 9- CUR+ORI 5 μM

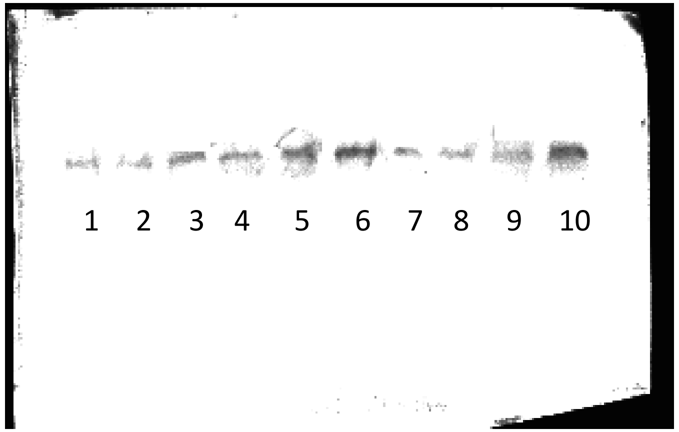

Figure S2. The representative immunoblot respectively for Figure 11.
The level of Caspase-3 protein in U-138 MG cell line.
1 - Control, 2 - Act 0,25 μM, 3 – Act 0,5 μM, 4- Act 1 μM, 5 - Act 5 μM, 6- Act 10 μM, 7 – CUR+ORI 0,5 μM, 8- CUR+ORI 1 μM, 9 - CUR+ORI 5 μM, 10- CUR+ORI 10 μM

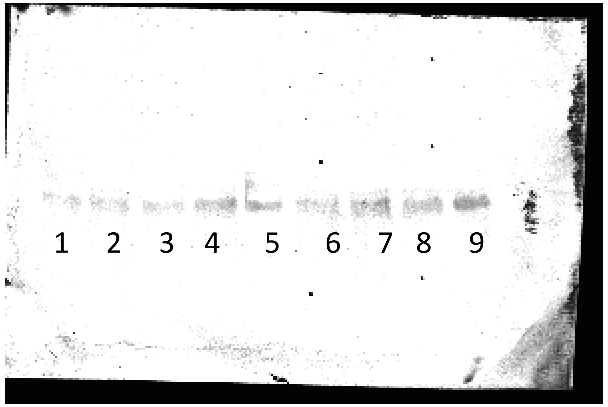

Figure S3. The representative immunoblot respectively for Figure 11.
The level of p53 protein in T98G cell line.
1 - Control, 2 - Act 0,25 μM, 3 – Act 0,5 μM, 4- Act 1 μM, 5 - Act 5 μM, 6 – CUR+ORI 0,25 μM,
7- CUR+ORI 0,5 μM, 8 - CUR+ORI 1 μM, 9- CUR+ORI 5 μM,

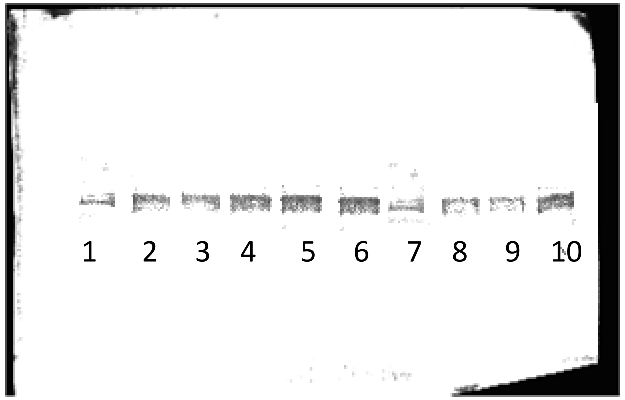

Figure S4. The representative immunoblot respectively for Figure 11.
The level of p53 protein in U-138MG cell line.
1 - Control, 2 - Act 0,25 µM, **3** – Act 0,5 µM, 4- Act 1 µM, 5 - Act 5 µM, 6- Act 10 µM,
7 – CUR+ORI 0,5 µM, 8- CUR+ORI 1 µM, 9 - CUR+ORI 5 µM, 10- CUR+ORI 10 µM,
